# Measuring cytokines in Eurasian tundra reindeer (*Rangifer tarandus tarandus*) with a bovine bead-based multiplex immunoassay and real-time PCR

**DOI:** 10.1186/s13028-025-00819-4

**Published:** 2025-06-18

**Authors:** Tuva Løken Frøvoll, Kari Lybeck, Hege Lund, Shokouh Makvandi-Nejad, Unni Grimholt, Carlos G. das Neves, Morten Tryland, Ingebjørg Helena Nymo, Siv Klevar

**Affiliations:** 1 Section for Virology, Immunology and Parasitology, Norwegian Veterinary Institute, 1433 Ås, Norway; 2 Faculty of Veterinary Medicine, Norwegian University of Life Sciences, 1433 Ås, Norway; 3 Fish Health Research, Norwegian Veterinary Institute, 1433 Ås, Norway; 4 Research, Education and Communication Section, Faculty of Health Sciences, UiT The Arctic University of Norway, 9019 Tromsø, Norway; 5 Department of Forestry and Wildlife Management, Inland Norway University of Applied Sciences, N-2480 Koppang, Norway; 6 Department of Arctic and Marine Biology, Faculty of Biosciences, Fisheries and Economics, UiT The Arctic University of Norway, 9019 Tromsø, Norway; 7 Research Group Food Safety and Animal Health, Norwegian Veterinary Institute, 9016 Tromsø, Norway

**Keywords:** Cervid herpesvirus, Cytokines, Gene expression, Mitogen stimulation, Orf virus, Parapoxvirus, Peripheral blood mononuclear cells (PBMCs), qPCR, Reindeer health, Varicellovirus cervidalpha2

## Abstract

**Background:**

Reindeer (*Rangifer tarandus tarandus*) herding is based on access to seasonal pastures. Pastureland is, however, being lost and fragmented due to e.g. climate change, human activities, and predators, creating an increasing need for feeding and fencing. This alters disease occurrence, leading to a greater need for disease investigation tools. Knowledge of the activation of immune pathways during disease can be obtained by measuring cytokines, but no commercial methods are currently available for reindeer. This study investigated whether the MILLIPLEX® Bovine Cytokine Magnetic Bead assay could be used to detect interleukin (IL)-6, IL-8, IL-10, IL-17, tumor necrosis factor-alpha (TNF-α), and interferon-gamma (IFN-γ) in reindeer cell supernatants and serum. Peripheral blood mononuclear cells (PBMCs) from reindeer (n = 4) and cattle (*Bos taurus*, n = 3) were stimulated with mitogens for 6 and 24 h (h) and the quantity of cytokines in cell supernatants was measured. Serum from experimental viral infections in reindeer (Orf virus; ORFV and Varicellovirus cervidalpha2; CvHV2) was also analysed. Additionally, primers were designed to measure cytokine gene expression in response to mitogens by real-time polymerase chain reaction (qPCR).

**Results:**

The bovine bead-based multiplex immunoassay detected five of six cytokines (IL-8, IL-10, IL-17, TNF-α, IFN-γ) in reindeer PBMC supernatants after stimulation. All cytokines were detected in bovine samples. Although cytokine concentrations were generally higher in bovine samples, analysis of reindeer supernatants demonstrated significantly increased IL-10, IL-17, TNF-α and IFN-γ concentrations in supernatants from stimulated compared to unstimulated PBMCs. Neither reindeer nor cattle samples showed a significant increase for IL-6, while IL-8 was increased only in bovine samples after 6 h stimulation. Serum from reindeer infected with CvHV2 showed significantly increased IFN-γ levels on days 4 and 7 post inoculation. Gene expression of all cytokines was increased by stimulation of reindeer PBMCs, except IL-6 for which primer design was unsuccessful.

**Conclusions:**

This study shows the potential of the bovine bead-based multiplex immunoassay for measuring IL-10, IL-17, TNF-α, and IFN-γ concentrations in reindeer. The qPCR is suitable for measuring gene expression of these cytokines and IL-8. These methods may be used to characterise immune responses in reindeer, but further testing and validation are warranted.

**Supplementary Information:**

The online version contains supplementary material available at 10.1186/s13028-025-00819-4.

## Background

Reindeer herding is practiced on 40% of the Norwegian land area and represents an important part of Sámi culture and employment in Sámi local communities [1]. Although Norwegian semi-domesticated reindeer (Eurasian tundra reindeer; *Rangifer tarandus tarandus*) are generally in good health, the fragmentation and loss of pastureland, as a result of among others a warmer and more unstable climate [2], human encroachment [2, 3] and predators [2, 4], has led to an increasing need for feeding and sometimes also fencing [5]. Feeding and fencing may be necessary to ensure reindeer survival during harsh winters; however, these practices can also influence disease occurrence, as noted in traditional Sámi knowledge [6] and in studies from Sweden and Finland, where reindeer are more extensively fed and fenced compared to Norway [5]. In addition, the reindeer can become stressed during feeding and fencing, which can trigger latent infections [7].

The Orf virus (ORFV), belonging to the *Parapoxvirus* genus within the *Poxviridae* family, is prevalent globally among sheep (*Ovis aries*) and goats (*Capra hircus*) [8]. In reindeer, ORFV manifests as contagious ecthyma, characterized by distinctive “cauliflower-like” growths at mucocutaneous junctions around the mouth and nose, and in the oral mucosa [9]. Incidences of contagious ecthyma outbreaks have been documented in reindeer in Norway, Sweden and Finland [10], and appeared as udder infections after calving in at least 22 females in 2016 in Iceland [11]. It also has zoonotic potential, inducing painful skin lesions in humans [12].

The reindeer alphaherpesvirus, known as Varicellovirus cervidalpha2 (CvHV2, formerly known as cervid herpesvirus 2), is endemic within the Fennoscandian reindeer population [13]. CvHV2-induced mucosal lesions in the eyes can predispose reindeer to secondary infections, potentially leading to infectious keratoconjunctivitis (IKC) [14]. This virus has been implicated in upper respiratory tract infections in reindeer and can be transmitted from mother to foetus [15]. Herpesviruses create life-long infections and latency, and disease outbreaks caused by CvHV2 are often associated with prior stress from herding, transportation and corralling [16, 17].

Measuring immune markers, such as cytokines, is essential to better understand the immune response to pathogens, and to gain a deeper insight into the immune pathways activated during an infection [18]. Common cytokine detection techniques include gene expression measurement with real-time polymerase chain reaction (qPCR) and antibody-based assays for protein measurement such as enzyme-linked immunosorbent assay (ELISA) or bead-based multiplex immunoassays. An advantage of bead-based multiplex immunoassays is that they allow the measurement of multiple analytes in a single sample run, reducing the sample handling and sample volume needed. Specific capture antibodies are coupled to fluorescence-barcoded Luminex magnetic beads for simultaneous detection of multiple cytokines [19]. Several multiplex immunoassays for detection of cytokines have been devised for humans, mice and rats, but there has been a scarcity of such methods for other species [20]. There are currently no commercial methods for the detection of cytokines in reindeer, but the commercial Cervigam interferon-gamma (IFN-γ) ELISA developed for red deer (*Cervus elaphus*) has been shown to detect reindeer IFN-γ [21]. The multiplex immunoassay MILLIPLEX® Bovine Cytokine Magnetic Bead assay has been evaluated for limits of detection, recovery rate, and reproducibility using plasma supernatants from stimulations of bovine whole blood [22]. Additionally, the MILLIPLEX® Bovine Cytokine Magnetic Bead assay has successfully been applied to other species, such as African buffalo (*Syncerus caffer*) [23], Mediterranean buffalo (*Bubalus bubalis*) [24] sheep [25], and goat [26]. However, no studies utilizing bead-based multiplex assays for reindeer are described in the literature. There are also few descriptions of specific primers for measuring cytokines in reindeer by qPCR [27], and information about reindeer cytokine protein-coding genes is not available in published databases.

The aim of the present study was to investigate whether the bovine bead-based multiplex immunoassay MILLIPLEX^®^ Bovine Cytokine Magnetic Bead assay could detect interleukin (IL)-6, IL-8, IL-10, IL-17, tumor necrosis factor-alpha (TNF-α), and IFN-γ in mitogen stimulated peripheral blood mononuclear cells (PBMCs) supernatants and serum samples from ORFV and CvHV2 infected reindeer. Additionally, specific primers were designed for reindeer exons, targeting IL-6, IL-8, IL-10, IL-17, TNF-α and beta-2-microglobulin (β2M) to measure cytokine expression at the mRNA level by qPCR.

## Methods

### Samples

Blood samples for stimulation of PBMCs were collected from healthy reindeer (n = 4) at UiT The Arctic University of Norway (UiT, Tromsø, Norway) and sent overnight to the Norwegian Veterinary Institute (NVI, Ås, Norway) where sample processing began the following day. Blood samples from healthy cattle (*Bos Taurus,* n = 3) were collected at the Norwegian University of Life Sciences (NMBU, Ås, Norway) and delivered immediately to NVI. All blood samples were taken from the jugular vein into ethylenediamine tetra-acetic acid (EDTA) tubes. The use of experimental animals was approved by the Norwegian Animal Research Authority (application identity numbers, FOTS ID, 24,386 and 29,626).

In addition, cytokine levels were measured in serum samples from two previous experimental infection trials on reindeer, with ORFV (2004; n = 6) [28] and CvHV2 (2009; n = 5) [29]. Healthy reindeer were inoculated with virus, and serum samples were collected at the inoculation day and days 5 and 12 post inoculation (p.i.) (ORFV) or days 0, 4, 7 and 10 p.i. (CvHV2). Day 0 samples were used as controls when measuring cytokine levels. In addition, among the animals from the infection trial with CvHV2, a non-inoculated control animal (Reindeer B1, designated as R1 in [29]) was included. This animal received sterile water intratracheally (i.e., treated as the other experimental animals, but not inoculated with CvHV2) and was kept within the same fenced area as the other animals. The control animal never showed any humoral immune response against CvHV2, indicating that no transmission from the inoculated animals had occurred [29]. Samples were shipped frozen to NVI and stored at -20 °C, until analysis.

### Isolation and stimulation of peripheral blood mononuclear cells

PBMCs were isolated on Lymphoprep density-gradient centrifugation medium (STEMCELL Technologies Inc., Vancouver, BC, Canada) and washed once in phosphate-buffered saline (PBS) containing 2 mM EDTA and twice in PBS without EDTA. Cells (2 × 10^6^ cells/mL) diluted in cell culture medium (Roswell Park Memorial Institute (RPMI) 1640) with Stable Glutamine (Biowest, Nuaillé, France) supplemented with 10% foetal bovine serum (Gibco BRL, Grand Island, NY, USA) and 1% Penicillin–Streptomycin (10,000 units penicillin and 10 mg streptomycin per mL in 0.9% NaCl, Sigma-Aldrich, St. Louis, MO, USA) were stimulated with 1X solution of Cell Stimulation Cocktail with phorbol myristate acetate (PMA; 50 ng/mL) and ionomycin (I; 1000 ng/mL) (PMA-I, eBioscience, Invitrogen; Thermo Fisher Scientific, San Diego, CA, USA) or media alone (unstimulated control) in 24-well cell culture plates (Corning Incorporated, Corning, NY, USA) for 6 and 24 h ( at 37 °C in humidified air with 5% CO_2_. PMA-I was selected for stimulation of PBMC, as low or variable cytokine levels had been detected during initial stimulations with phytohaemagglutinin (PHA), concanavalin A (ConA), or lipopolysaccharide (LPS) (Additional file 1). After 24 h of stimulation, cell culture supernatants were harvested and stored at −80 °C until analysis.

### Bead-based multiplex cytokine analysis

Cytokine levels for IL-6, IL-8, IL-10, IL-17, TNF-α, and IFN-γ were measured using a bovine bead-based multiplex immunoassay (MILLIPLEX^®^ Bovine Cytokine/Chemokine Magnetic Bead multiplex assay kit, Merck Millipore, Darmstadt, Germany).

This multiplex assay is available as a 15-plex kit, including IFN-γ, IL-1α, IL-1β, IL-4, IL-6, IL-8, IL-10, IL-17A, macrophage inflammatory protein (MIP)-1α and MIP-1β, interleukin 36 receptor antagonist (IL-36RA), IFN-γ-inducible protein 10 (IP-10), monocyte chemoattractant protein 1 (MCP-1), TNFα, and vascular endothelial growth factor A (VEGF-A). Alternatively, you can select only the cytokines of interest. For the current study, it was decided to include IL-6, IL-8, IL-10, IL-17, TNF-α, and IFN-γ to limit the experiment’s size while still covering cytokines related to both innate and adaptive immunity, as well as pro- and anti-inflammatory responses. The multiplex kit was provided with separate bead vials (not custom premixed), and the assay was performed following the manufacturer’s instructions. Samples were thawed, mixed and centrifuged. PBMC supernatants from bovine and reindeer samples were analysed undiluted in biological duplicates, while serum was diluted 1:2 in assay buffer and analysed in technical duplicates. The magnetic beads were sonicated (30 s) and vortexed (1 min) before transferring 60 μL of each bead to a mixing bottle, adding assay buffer for a final volume of 3000 μL and vortexing. The 96-well plate was added 200 µL wash buffer, incubated on a plate shaker for 10 min at room temperature (RT) before decanting the wash buffer. Next, 25 μL standards, quality controls, or assay buffer only (background) were applied to the 96-well plate. Assay buffer (25 μL) was further added to samples wells, and 25 μL of appropriate matrix solution was applied to the background, standards, and control wells (kit serum matrix for analysing serum samples, and assay buffer for analysing cell culture supernatants), before adding 25 μL samples to sample wells. Thereafter, 25 μL of the magnetic bead mix was added to all wells and the plate sealed, covered with a light-proof lid, and incubated on a shaker at RT for 2 h. The plate was subsequently washed three times and 25 μL of detection antibody was applied per well. Following 1 h incubation as above, 25 μL streptavidin–phycoerythrin (SA-PE) was added to each well. The plate was incubated for another 30 min as previously described, washed three times and added 150 μL of sheath fluid. The plate was shaken for 5 min before analysis. The median fluorescent intensity (MFI) of each well was obtained using the Bio-Plex 200 (Bio-Rad Laboratories, Alcobendas, Spain) system and data were processed using BioPlex Manager 6.2 (Bio-Rad). It was ensured that standards and quality controls remained within their tabulated values. Coefficient of variation (%CV) was calculated for each duplicate ([standard deviation (SD) / mean] × 100). Standard curve outliers were excluded by the software if situated outside the standard’s acceptable recovery range (70–130%). Sample duplicates were manually excluded where the CV exceeded 30%, resulting in the exclusion of 4 low out of range values. The CV for technical duplicates was below 30% for all analysed samples. Samples above or below limit of quantification (LOQ) were replaced with LOQ values and are shown as open symbols in result figures. During the analysis of serum samples from infection trials, the quality controls failed, showing concentrations lower than the reference values provided by the manufacturer. This was consistent for all cytokines; therefore, cytokine levels in serum from infection trials are reported as MFI instead of concentrations [30].

### Sequence mining

The amino acid sequences of bovine cytokines were acquired from Uniprot [30] while those from reindeer were identified using tblastN of cattle amino acid sequences against the reindeer genome (GCA_902712895.1). The coding nucleotide sequences (exons) of the genes were determined using the Basic Local Alignment Search Tool (BLAST) software [32]. Specifically, cattle exon sequences were aligned against the reindeer genome (GCA_902712895.1) in the National Center for Biotechnology Information (NCBI) database. The nucleotide sequence (exon) for IL-17 (NM_002190.3) was obtained from the Yarkand deer (*Cervus hanglu yarkandensis*, GCA_010411085.1) in the Ensembl database [33]. To verify homology, multiple sequence alignment (MSA) was conducted on IL-6, IL-8, IL-10, IL-17, TNF-α, and IFN-γ sequences from reindeer and cattle using Unipro Ugene [29]**,** employing the Multiple sequence alignment and fast Fourier transform (MAFFT) algorithm.

### Primer design

Specific primer sequences for IFN-γ in reindeer were obtained from a previous study [27]. Specific primers for IL-6, IL-8, IL-10, IL-17, and TNF-α target genes and the normalizing β2M reference gene were designed using Primer 3 version 4.1.0 software [31]. The primer sequences and expected PCR product lengths are shown in Table 1. Primers were designed based on exon sequence conservation and were checked against the reindeer genome to verify the exon–intron structure. Primer sequences were chosen to prevent reactivity to other coding sequences. The primers were synthesized and supplied by Eurogentec (Liege, Belgium).

**Table 1:** Primers for reindeer (*Rangifer tarandus tarandus*) cytokines

Target^a^	Oligonucleotide sequences (5’ → 3’)^a^	Product size (bp)	PCR efficiency (%) ^b^	References
IL-6	F: CTCTTCACAAGCGCCTTCAG	120	-	This study
	R: GCTTGGGGTGGTGTCATTTT			
IL-8	F: AACACATTCCACGCCTTTCC	118	105	This study
	R: GCAGACCTCTTTTCCGTTGG			
IL-10	F: AACCATGGGCCTGAGATCAA	119	107	This study
	R: ACCGCCTTGCTCTTGTTTTC			
IL-17	F: GGTACCCCTCTGTGATCTGG	109	100	This study
	R: AGGATCTCTTGCTGGATGGT			
TNF-α	F: CCAAAAGCATGATCCGGGAC	111	100	This study
	R: GGAGGAAGGAGAAGAGGCTG			
IFN-γ	F: GCGCAAAGCCATAAATGAAC	98	101	[27]
	R: CTTCTCTTCCGCTTTCTGAG			
β2M	F: GGATGGGAAGCCAAATCACC	150	99	This study
	R: TGGGACAGCAGGTAGAAAGA			

^a^Specific oligonucleotide sequences designed to detect interleukin (IL)-6, IL-8, IL-10, IL-17, tumor necrosis factor-alpha (TNF-α) and beta-2-microglobulin (β2M) exons in samples from reindeer (*Rangifer tarandus tarandus*), GenBank accession number GCA_902712895.1) using the NCBI Genomes database. Primers targeting interferon-gamma (IFN-γ) were described in a previous study [27]. F, forward primer; R, reverse primer

^b^PCR efficiency (E) = (10 ^−1/slope^ − 1) × 100

Gel electrophoresis was performed to confirm that the primers produced PCR products of the expected size. PCR was conducted with cDNA (0.8 μg/mL) using the KAPA HiFi HotStart ReadyMix kit (Roche, Basel, Switzerland) and a T100TM Thermal Cycler (Bio-Rad, California, USA) under the following conditions: 95 °C for 3 min, 98 °C for 20 s followed by 35 cycles at 60 °C for 15 s, then 72 °C for 20 s, and finally 72 °C for 1 min. The PCR products were analysed by 2% agarose gel electrophoresis (110 V, 1 h) and visualized using the Azurec150 Gel Imaging System (Azure Biosystems, California, USA).

Primer efficiencies (E) were assessed by conducting a tenfold dilution series of purified target gene products in duplicate. The quality of each standard curve was evaluated based on the slope. The slope of the line derived from the dilution series was employed to calculate the efficiency of target amplification using the formula E = (10^(-1/slope)—1) × 100, with 100% indicating optimal efficiency. The resultant PCR products were subjected to Sanger sequencing and analysed in Sequencher 5.4.6 (Gene Codes, Ann Arbor, MI, USA).

### RNA extraction

Total RNA was extracted from PBMCs using the RNeasy Mini Kit (Qiagen, Hilden, Germany) in combination with QIAshredder spin columns (Qiagen) and DNase treatment (RNase-Free DNase Set, Qiagen). To increase RNA yield, a washing step with PBS was included in the protocol before adding the RLT buffer. Concentration (µg/mL) and purity of the RNA was measured using mySPEC spectrophotometer (VWR, Leuven, Belgium). Extracted total RNA was stored at -80 °C until further use.

### Complementary DNA synthesis

Diluted total RNA (20 µg/mL) was reverse transcribed into complementary DNA (cDNA) using the QuantiTect^®^ Reverse Transcription Kit (Qiagen) and Veriti 96-Well Fast Thermal Cycler (Thermo Fisher Scientific) including a genomic DNA elimination reaction following the manufacturer's protocol. RNA samples (n = 7) with initial concentration < 20 µg/mL were used undiluted, and to adjust for this, water was not added during the reverse transcription of RNA. The cDNA was stored at −20 °C until further use.

### Real-time PCR (qPCR)

The qPCRs were carried out in 20 μL volumes and performed using a CFX96 Touch Real-Time PCR system (Bio-Rad) programmed in Bio-Rad CFX Manager 3.1. The PCR mixture contained 2 μL of 1:5 diluted cDNA, 10 μL 2 × SsoAdvanced Universal SYBR Green Supermix (Bio-Rad), 0.5 μL 10 μM forward and reverse primers, and 7 μL RNase-free water. The plate was sealed using the PX1 PCR Plate Sealer (Bio-Rad), shaken for 30 s on an IKA MS3 Mini-shaker (Millipore Sigma, Staufen, Germany), and then spun on a PCR plate spinner (VWR). Each sample was analysed in duplicate. Thermal cycling conditions were 95 °C for 30 s, followed by 40 cycles with 95 °C for 15 s, 60 °C for 30 s, and 55 °C for 5 s.

Relative gene expression was calculated in Microsoft Office Excel, using the ΔΔCq method [31]. Values were expressed relative to the unstimulated control sample and the reference gene was β2M. All samples are reported as relative transcription or the *n*-fold difference between the reference unstimulated sample and the mitogen stimulated samples.

### Statistical analysis

Descriptive plots were used to give an overview of individual cytokine measurements and summary statistics (median) for data grouped by species, treatment group (stimulated versus unstimulated) and time since stimulation. Similar plots for the infection trials included a line between the MFIs measured from the same individuals.

In order to test for i) effect of stimulation and differences in cytokine levels between ii) species and iii) time since stimulation, we fitted a generalized linear model (*glm*) using a gamma distribution with a log-link function to stabilise the variance. For each cytokine, the concentrations were fitted as a function of species and treatment group (stimulated at 6 h, unstimulated at 6 h, stimulated at 24 h, unstimulated at 24 h), and the interaction term between species and treatment groups. For cytokines IL-6 and IL-8, the regression model was fitted to reindeer and/or cattle separately. Differences between unstimulated and stimulated PBMCs were considered significant if P < 0.05.

Cytokine levels (MFI) in serum from infection trials were analysed by a regression analysis for each cytokine as a function of group according to days p.i. Random effect intercepts were included to account for the different levels of each individual, using the lme function in the R package *nlme* [32]. An indicator variable was included for the control (Reindeer B1).

Statistical analyses were conducted in R version 3.6.1 [33].

## Results

### High amino acid homology between reindeer and cattle cytokines

MSA indicated high amino acid homology between reindeer and cattle cytokines. The sequences for IL-6, IL-8, IL-17, and TNF-α were found to be homologous between reindeer and cattle. IL-10 differs at positions 51 and 52; here reindeer has glutamic acid and alanine, whereas cattle have lysine and valine. IFN-γ differs in position 166; in this position reindeer has threonine and cattle has methionine (Additional file 2).

### Bovine bead-based multiplex immunoassay demonstrated feasibility in measuring reindeer cytokines

**Fig. 1:**
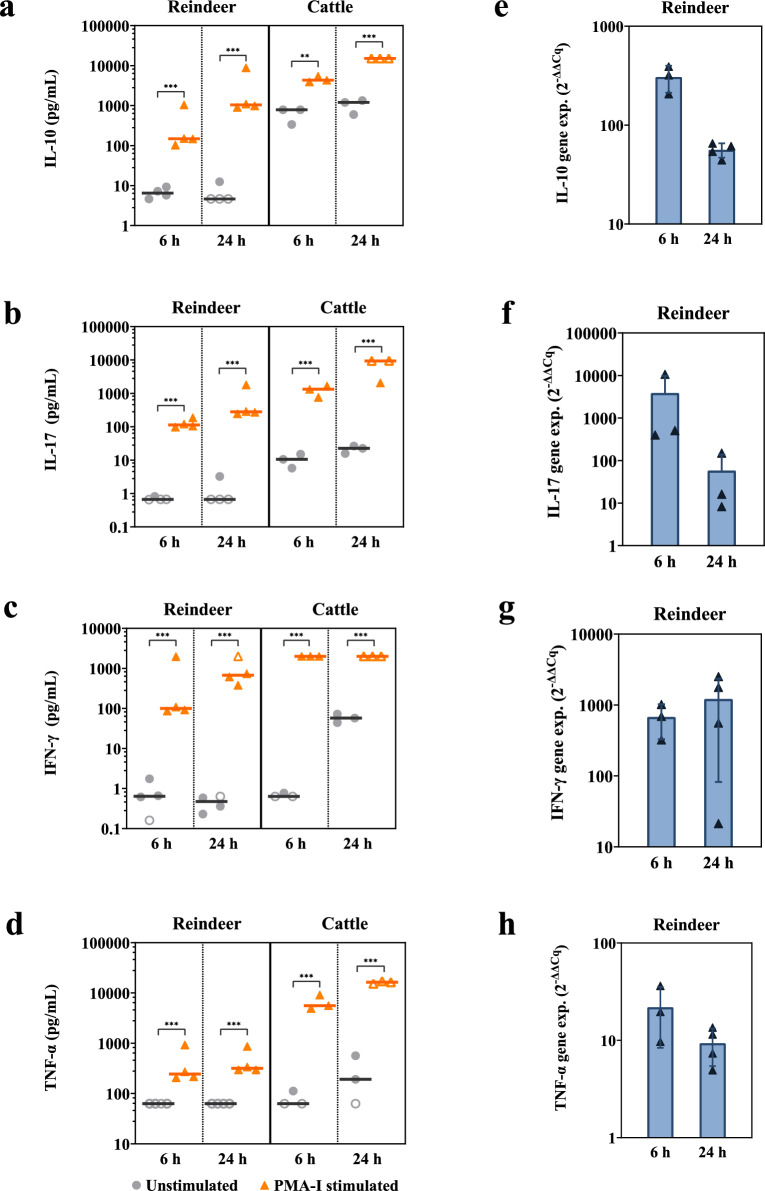
Protein concentrations and relative gene expression levels of cytokines after mitogen stimulation of PBMCs. **(a-d)** Protein concentrations in supernatants from unstimulated control and phorbol myristate acetate and ionomycin (PMA-I) stimulated peripheral blood mononuclear cells (PBMCs), measured using MILLIPLEX^®^ Bovine Cytokine/Chemokine multiplex assay**.** The x-axis represents stimulation time in hours (h), with reindeer data (n = 4) on the left and cattle data (n = 3) on the right. Vertical lines indicate median protein concentration (pg/mL). Points denote individual measurements for unstimulated control (●) and PMA-I incubated (▲) samples. Open symbols (○, ∆) indicate points higher or lower than the limits of quantification (LOQ). **(e–h)** Relative gene expression levels after mitogen stimulation of PBMC. The x-axis represents stimulation time in hours (h) for reindeer PBMCs (n = 3–4). Bars depict averages ± standard deviation (SD) of relative mRNA levels, expressed as fold change. Points denote the fold change for individual animals (▲). Calculations utilize the 2^−ΔΔCq^ method, with β2M in unstimulated PBMCs as a reference. Significantly different from unstimulated samples: *** P < 0.001, ** P < 0.01

**Fig. 2:**
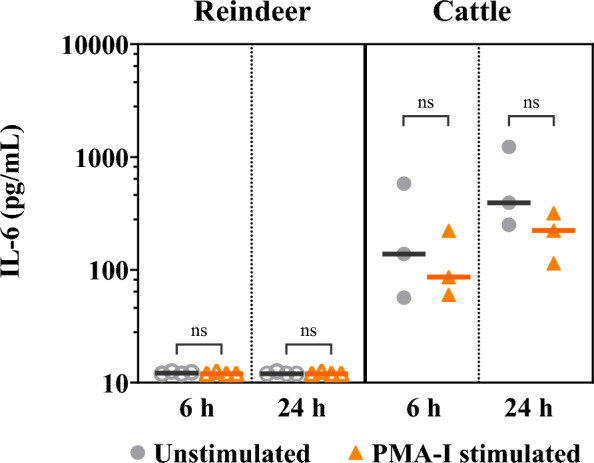
IL-6 concentration after mitogen stimulation of PBMCs. IL-6 concentrations in supernatants from unstimulated controls and phorbol myristate acetate and ionomycin (PMA-I) stimulated peripheral blood mononuclear cells (PBMCs) measured using MILLIPLEX® Bovine Cytokine/Chemokine multiplex assay. The x-axis represents stimulation time in hours (h), with reindeer data (n = 4) on the left and cattle data (n = 3) on the right. Vertical lines indicates median protein concentration (pg/mL) Points denote individual measurements for unstimulated control (●) and PMA-I incubated (▲) samples. Open symbols (○, ∆) indicate points higher or lower than the limits of quantification (LOQ). Not significantly (ns) different from unstimulated samples (P < 0.05)

**Fig. 3:**
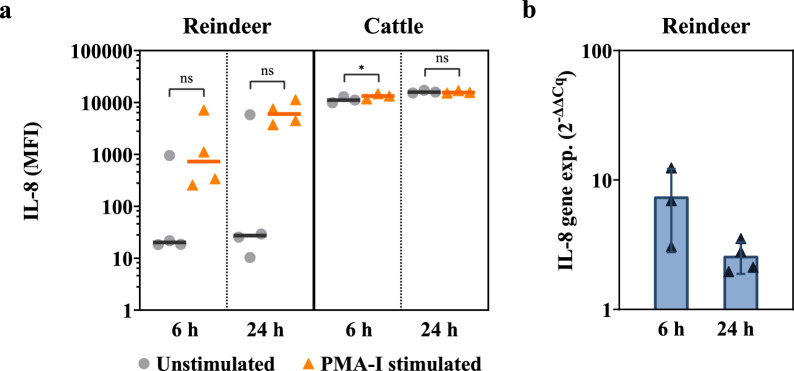
Protein levels and relative gene expression levels of IL-8 after mitogen stimulation of PBMCs. **(a)** IL-8 median fluorescent intensity (MFI) in supernatants from unstimulated and phorbol myristate acetate and ionomycin (PMA-I) stimulated peripheral blood mononuclear cells (PBMCs), measured using MILLIPLEX® Bovine Cytokine/Chemokine multiplex assay. The x-axis represents stimulation time in hours (h), with reindeer data (n = 4) on the left and cattle data (n = 3) on the right. Vertical lines indicate median MFI in supernatants. Points denote individual measurements for unstimulated control (●) and PMA-I incubated (▲) samples. Significantly different from unstimulated samples: * P = 0.05. ns: Not significantly different from unstimulated samples (P < 0.05). The kit-specific standards used to generate a 7-point standard curve often deviated from the recovery range, results are therefore reported as MFI and not converted to concentration (pg/mL). **(b)** Relative gene expression levels after mitogen stimulation of PBMC. Bars depict averages ± standard deviation (SD) of relative mRNA levels, expressed as fold change. Points denote the fold change for individual animals (▲). Calculations utilize the 2^−ΔΔCq^ method, with β2M in unstimulated PBMCs as a reference

The bovine bead-based multiplex immunoassay detected measurable concentrations for five of the six cytokines in the supernatant of PMA-I stimulated reindeer PBMCs, namely IL-10, IL-17, TNF-α and IFN-γ (Fig. 1a–d), and IL-8 (Fig. 3). Cytokine levels measured in reindeer were overall lower than in cattle. For both reindeer and cattle, supernatants of stimulated PBMCs showed significantly increased concentrations of IL-10 (P < 0.001, for all except P = 0.006 for cattle at 6 h), IL-17 (P < 0.001), TNF-α (P < 0.001) and IFN-γ (P < 0.001) compared to supernatants of unstimulated cells (Fig. 1a–d). Concentrations were higher 24 h after stimulation compared to 6 h for IL-10 (P = 0.001 for reindeer, P = 0.07 for cattle), IL-17 (P < 0.01) and TNF-α for cattle (P = 0.05) (Fig. 1a–d). The multiplex assay did not detect measurable levels of IL-6 in reindeer. For cattle however, IL-6 was detectable, but the concentration did not change significantly after mitogen stimulation (Fig. 2). Initial stimulations of reindeer PBMC with PHA, ConA or LPS had been performed, but IL-6 was not detected during these conditions either (Additional file 1).

Stimulation did not induce a significant increase in IL-8 levels in PBMC-derived supernatants from neither reindeer nor cattle, compared to controls after 24 h stimulation. For cattle at 6 h, the IL-8 levels were slightly higher in stimulated cells compared to controls (P = 0.05) (Fig. 3). Despite the detection of high IL-8 levels in both reindeer and cattle samples, the kit-specific standards used to generate a 7-point standard curve often deviated from the recovery range, and hence the measured MFI could not consistently be converted to concentrations.

**Fig. 4:**
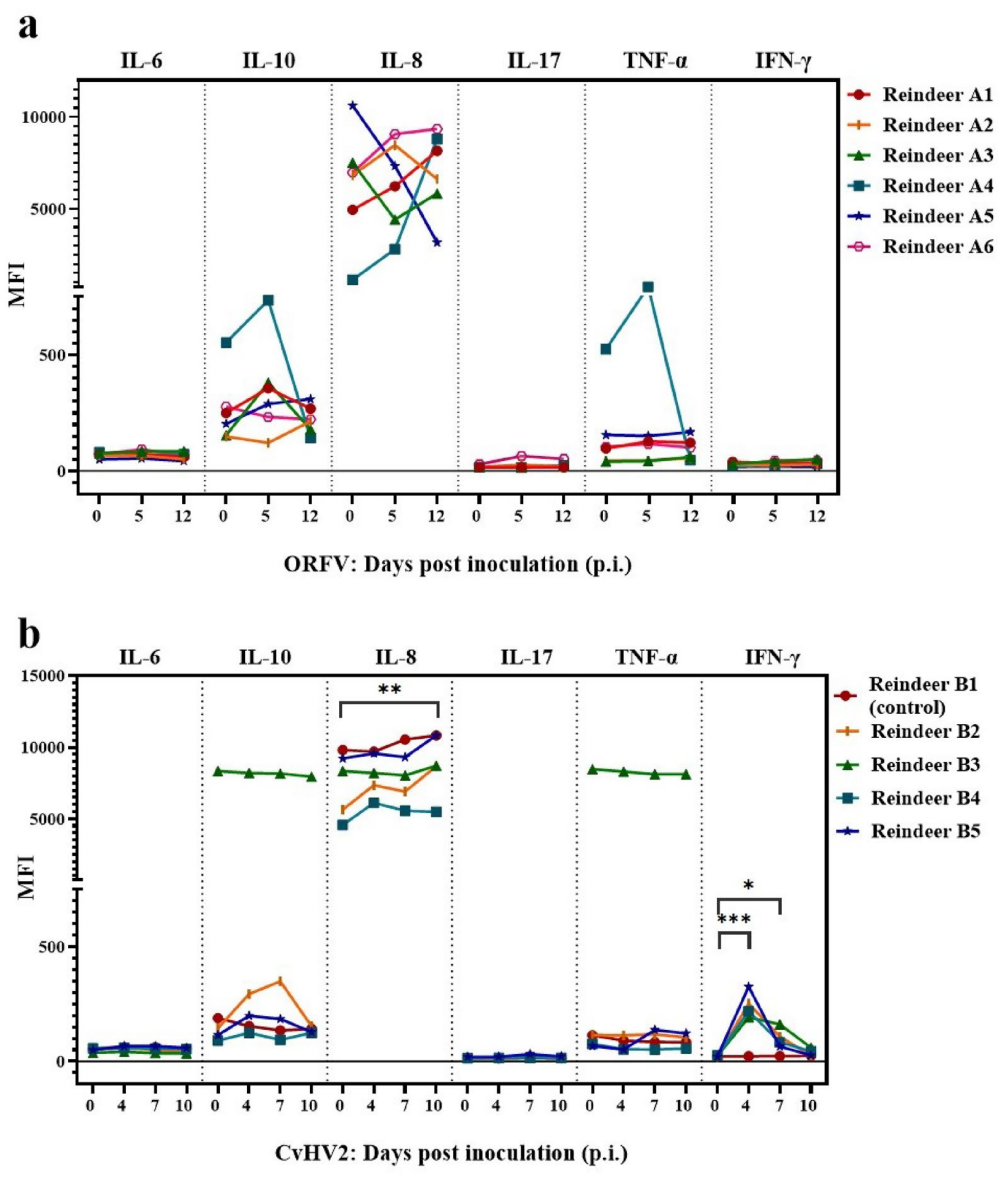
Cytokine levels measured in serum samples from reindeer inoculated with ORFV or CvHV2. Quality control samples showed concentrations lower than the expected table values provided by the manufacturer, and results are therefore reported as median fluorescent intensity (MFI) and not converted to concentration (pg/mL). **(a)** Cytokine levels measured in serum samples from reindeer (n = 6) 0, 5, and 12 days post inoculation (p.i.) with Orf virus (ORFV) [28] using the MILLIPLEX^®^ Bovine Cytokine/Chemokine multiplex assay. The different days of sampling are indicated for each cytokine. **(b)** Cytokine levels measured in serum samples from reindeer (n = 5) 0, 4, 7, and 10 days p.i. with Varicellovirus cervidalpha2 (CvHV2) [29] using the MILLIPLEX® Bovine Cytokine/Chemokine multiplex assay. Reindeer B1 is a non-inoculated control animal. The different days of sampling are indicated for each cytokine. Significantly different from unstimulated samples: *** P < 0.001, ** P < 0.01, * P = 0.01

Serum samples from reindeer inoculated with ORFV [28] showed low levels of IL-6, IL-17, and IFN-γ at all times p.i. (Fig. 4a). Levels of IL-10 and IL-8 shifted after ORFV inoculation, but no significant differences were detected as individual values changed in different directions. One animal stood out with higher levels of IL-10 and TNF-α which increased at day 5 p.i., followed by a decrease (Reindeer A4). All animals had high IL-8 levels at all times p.i. (Fig. 4a).

A significant increase in IFN-γ levels on days 4 (P < 0.001) and 7 (P = 0.01) p.i. was observed in serum samples collected from reindeer inoculated with CvHV2 [29] compared to day 0 (Fig. 4b). The level at day 4 was also significantly higher than for day 7 and 10 (P < 0.001). The same increase was not seen in the negative control animal (Reindeer B1). Serum levels of IL-6 and IL-17 remained low at all times p.i. IL-10 and TNF-α levels were not significantly affected after CvHV2 inoculation compared to day 0, although one individual (reindeer B3) exhibited higher levels of IL-10 and TNF-α compared to the other animals at all times p.i. All animals had high IL-8 levels throughout the study, and at day 10 p.i. the level was higher (P = 0.002) compared to day 0.

### qPCR effectively measures cytokine gene expression in reindeer

Gene expression of IL-8, IL-10, IL-17, IFN-γ, and TNF-α was increased by PMA-I stimulation. The lowest mean relative gene expressions were found for IL-8 and TNF-α. Except for IFN-γ, the highest mean cytokine expression was detected after 6 h of stimulation (Fig. 1e–h, Fig. 3b).

The A_260/280_ ratio of the isolated RNA fell within the range of 1.8—2.0, indicating that the RNA was relatively free of proteins and contaminants. Gel electrophoresis showed bands of the expected size for IL-8, IL-10, IL-17, IFN-γ, and TNF-α (Additional file 3). PCR efficiencies and primers used for qPCR analysis of reindeer IL-8, IL-10, IL-17, IFN-γ, TNF-α, and β2M are shown in Table 1. PCR efficiencies were 99–107%, falling within the optimal interval of 90–110% [34]. Sequencing PCR products was successful, with the exception of IL-17. The attempt to design specific primers targeting IL-6 in reindeer proved unsuccessful, as gel electrophoresis indicated the presence of several bands.

## Discussion

The results of this study suggest that the commercial bovine cytokine multiplex immunoassay MILLIPLEX® Bovine Cytokine/Chemokine Magnetic Bead assay can be used to measure IL-10, IL-17, IFN-γ and TNF-α in in vitro activated reindeer PBMCs and reindeer serum. Furthermore, qPCR with the designed primers was shown to work effectively for measuring IL-8, IL-10, IL-17, IFN-γ, and TNF-α in reindeer samples. This implies that both the multiplex assay and the qPCR show potential as complementing tools for characterising immune responses in reindeer.

The bovine multiplex immunoassay detected five of six cytokines in reindeer samples indicating that the kit antibodies were able to bind reindeer cytokines. Our results are in agreement with other studies. Plasma from stimulated whole blood of African buffalo has yielded measurable concentrations for fifteen cytokines and chemokines, using the same bovine bead-based multiplex immunoassay [23]. In sheep, the method was successfully used for measuring cytokine levels in serum from LPS-injected lambs [25], while in goats it was successfully used for measuring cytokine levels in serum samples from a herd infected with *Mycobacterium bovis* [26]. The assay was also employed to identify cytokines that could potentially serve as biomarkers for bovine tuberculosis in Mediterranean buffalo [24]. Although the bovine bead-based multiplex immunoassay detected five cytokines, the cytokine levels in supernatants from PMA-I stimulated reindeer PBMCs were generally lower compared to levels in bovine samples. One reason for this could be that bovine samples were stimulated on the day of blood sampling, while reindeer blood was, due to logistics, transported to the laboratory and stimulated the next day. This could have affected the cells` ability to produce cytokines [35, 36]. Alternatively, it might be that the antibodies produced for detection of bovine cytokines bound less effectively to reindeer cytokines. When the bovine bead-based multiplex immunoassay was used to analyse plasma from African buffalos all cytokines were detected, but also at lower levels compared to bovine samples [23]. MSA revealed high degree of homology between reindeer and cattle amino acid sequences of the investigated cytokines, supporting the use of the commercial bovine assay for quantifying these cytokines in reindeer. Furthermore, species cross-reactivity of primers amplifying cytokine target genes in reindeer and cattle has previously been demonstrated [27]. Although there is a correlation between sequence homology and antibody cross-reactivity, high predicted sequence similarity at the mRNA or amino acid level does not always translate into high degree of antibody cross-reactivity. For instance, antibody binding may be affected by post-translational modifications of the target proteins. Additionally, linear peptides are often used as immunogens for antibody production, and antibody binding will depend on whether it recognizes the desired epitope on the molecular surface of the native protein [37, 38]. Nevertheless, the bovine bead-based multiplex immunoassay appears promising for measuring reindeer cytokines, but analysis of more samples is warranted. Further, as only a selection of the cytokines available for the MILLIPLEX® Bovine Cytokine/Chemokine multiplex assay was examined, future studies could also include testing the remaining cytokines and chemokines (IL-1α, IL-1β, IL-4, MIP-1α, IL-36RA, IP-10, MCP-1, MIP-1β, TNFα, VEGF-A).

The multiplex assay detected IL-8 in reindeer PBMC supernatants, but there were challenges in converting measured MFI to concentration values as the kit-specific standards used to generate the standard curve for IL-8 often deviated from the acceptable recovery range. Stimulation did not lead to a significant increase in IL-8 levels compared to controls. The exception was a slight increase in supernatants from bovine PBMCs after 6 h of stimulation, although IL-8 levels were also high in unstimulated PBMCs. Additionally, samples from reindeer in the ORFV and CvHV2 experiments had high IL-8 levels at all time points. A study evaluating the MILLIPLEX® Bovine Cytokine Magnetic Bead assay for use with bovine plasma following stimulation with bacteria and toll-like (TLR) receptor ligands reported that a significant number of the IL-8 values fell outside the assays quantification range [22]. Another study using the same bovine bead-based multiplex immunoassay for goats [26] observed high levels of IL-8 in serum samples from animals in both the control group and the group treated with two anti-inflammatory substances. Since all goats came from a *M. bovis*-infected herd the authors suggested that this was due to a high basal proinflammatory response to the infection. Although this might have been the case in the goat study, our results suggests that high basal levels of IL-8 in both reindeer and cattle samples need to be considered when studying this cytokine. In the current study, IL-8 levels are presented as MFI, as it has previously been reported that analysing MFI, rather than concentration values, would be acceptable for bead-based multiplex immunoassay data [30]. A previous study generally found comparable results, regardless of whether concentration or MFI values were used to define stimulation-related signatures, although small differences were detected for IL-8 [22]. They also reported that applying the upper limit of quantification to values exceeding the quantification range could falsely decrease the IL-8 variance and argued that retaining MFI values preserved greater variability and improved discrimination between different stimulation conditions. The main aim of the current study was to determine if the various cytokines could be detected, which was confirmed for IL-8. Dilution of samples with saturating levels of IL-8 should be explored in future studies to better distinguish between stimulated and unstimulated conditions. However, this approach may cause some samples to fall below the detection threshold for other cytokines measured in the same multiplex assay.

IL-6 was undetectable in both PBMC supernatants and serum samples from reindeer. It is uncertain whether the lack of IL-6 detection in supernatants is due to the PMA-I stimulation not effectively inducing IL-6 production or that the bovine anti-IL-6 antibody in the multiplex assay was unable to bind reindeer IL-6. As already mentioned, high predicted sequence similarity at the mRNA or amino acid level does not necessarily result in a high degree of antibody cross-reactivity. PMA-I has previously induced IL-6 production in sheep PBMC after overnight stimulation [39]. In the current study, however, only low IL-6 levels were detected in bovine supernatant with the multiplex assay, suggesting that the stimulation may not have been optimal for IL-6 activation. IL-6 was also not detected when reindeer PBMCs were stimulated with other mitogens during initial studies. Notably, stimulation of whole blood from cattle with TLR 2 and 4 ligands or heat-killed bacteria known to cause bovine mastitis, resulted in increased IL-6 levels compared to unstimulated controls [22]. Commercial reagents primarily produced for studies with human cells might not stimulate the innate immune response in ruminants in an optimal manner. Thus, additional serum samples from infected animals and supernatants from cells stimulated under different conditions should be analysed before concluding that the bovine bead-based multiplex immunoassay is unsuitable for detecting IL-6 in reindeer.

Among the two experimental infections, a notable change was observed solely in serum samples from reindeer infected with CvHV2, and only for IFN-γ and IL-8. There was a significant increase in IFN-γ levels on days 4 and 7 compared to day 0. This change was not observed in the control animal. IFN-γ is of critical importance for combating viral infections and its antiviral mechanisms against herpes simplex virus (HSV) and equine herpesvirus 1 (EHV-1) has been shown to include interference with viral gene expression and inhibition of viral replication [40, 41]. In humans, macrophages play an important role in the early defence against HSV. Initially, cytokines like type I IFN, and TNF-α are released, directly combating the virus and activating macrophages. Subsequently, IL-12, alongside other cytokines, prompts the production of IFN-γ in activated natural killer (NK) cells and T cells, reinforcing the anti-viral response [42]. The elevated IFN-γ levels on day 4 and 7 hence align with observations described in humans infected with HSV.

The serum analysis focused on samples collected at early time points after reindeer were infected with ORFV (0–12 days p.i.) or CvHV2 (0–10 days p.i.), as it was deemed most likely that any changes in cytokine levels would be detected during this period. With the exception of serum samples collected on day 0, serum samples were collected from animals showing clinical signs. Studies have shown that cytokines from humans stored at -80 °C remain stable for two years [43], but levels decrease after 8 days at -20 °C [44]. Serum from experimental infections with reindeer is not easily obtainable, and therefore serum that had been subjected to long-term storage was used in the current study. The limited changes in cytokine levels detected in reindeer serum after experimental infection may therefore be due to long-term storage at -20 °C resulting in cytokine degradation, and it would have been better to analyse serum after short-term storage at -80 °C.

Few studies describe the analysis of cytokines at the protein level in serum of animals during the course of virus infections. Circulating cytokines have been measured in serum using ELISA during African swine fever virus (ASFV) infection trials [45]. Despite acute, severe infection, IFN-γ was not detectable, possibly due to an impaired T-cell response caused by the ASFV [45]. In the current study, IFN-γ was not detected after ORFV infection, but levels increased significantly after CvHV2 infection. In the pig ASFV trial, levels of pro-inflammatory cytokines, including IL-6 and IL-8 increased from 3 days p.i., while IL-10 was only detected at the terminal stages of the infection together with a second peak in pro-inflammatory cytokine levels. In the current study, TNF-α, IL-8, and IL-10 showed no clear pattern or increase after infection in the ORFV and CvHV2 infection trials, except for an increase in IL-8 levels at day 10 p.i. in animals infected with CvHV2. IL-6 was not detected in serum samples, mirroring findings in cell supernatants. In a study examining the cytokine response to infection with bluetongue virus (BTV) serotype 1 or 8 in sheep, serum concentrations of IFN-γ did not show significant changes during the course of the experiment, although levels tended to be higher in sheep infected with BTV-1. A significant increase in IL-10 was detected 12 days p.i. in sheep infected with BTV-8, while TNF-α increased significantly 4 days p.i. in the BTV-1 group [46]. Overall, the type of infection, including severity and degree of inflammation, likely influence which cytokines are detected. Furthermore, studies analysing cytokines post-virus infection indicate the importance of timing for cytokine detection. Cytokines are potent mediators that act at low concentrations, primarily on nearby cells, and are rapidly cleared from the circulation [47]. Therefore, sampling at the right time to capture any increase in cytokine production post-infection can be challenging. Ideally, multiple time points post-infection should be analysed.

To facilitate the examination of cytokine levels in reindeer at the mRNA level, primers against IL-8, IL-10, IL-17, TNF-α, IFN-γ and β2M were utilized to measure gene expression using qPCR. The cytokine mRNA sequences for reindeer were unknown, thus, bovine sequences were applied to design reindeer cytokine primers [27]. IFN-γ primers used were optimized and validated to measure cytokine gene expression of red deer and cervid species in a previous study [27]. These primers were designed using conserved regions of cDNA, and could react with other species including reindeer, cattle, sheep, goat, white-tailed deer (*Odocoileus virginianus*), and bison (*Bison bison*) [27]. The results indicated that qPCR with the primers used in the current study, could successfully measure IL-8, IL-10, IL-17, IFN-γ, and TNF-α in reindeer samples. Sequencing of the IL-17 PCR product was unsuccessful. Despite convincing qPCR results and a band of the expected size by gel electrophoreses, sequencing of the IL-17 PCR product should be repeated before including these primers in other studies, to ensure that the correct sequence is amplified. Designing specific primers targeting IL-6 gene expression was not successful. β2M was selected as reference gene based on financial constraints and stabile expression in previous studies with stimulation of reindeer PBMC [48]. More reference genes should be evaluated and a combination of the genes found to be most stable during the experimental setting included in future studies measuring reindeer cytokine expression by qPCR [49].

By including both the multiplex assay and qPCR, it was possible to determine if mitogen stimulation resulted in increased cytokine production at the mRNA and protein level and at which time point. Cytokine levels of IL-10, IL-17, TNF-α, and IFN-γ inreased upon PMA-I stimulation for both assays. As previously described [50], relative cellular protein levels depends on mRNA abundance, but the transcript levels alone are not enough to predict protein levels. Additionally, early time points are preferable for mRNA quantification, whereas later time points are better for assessing protein concentrations of cytokines after cell stimulation. A previous study found that it was approximately a 5 h delay between IL-8 and TNF-α gene expression and the peak of the protein levels [51]. A similar pattern was found in the current study; the mean cytokine mRNA expression was generally highest after 6 h of stimulation, while protein levels often peaked after 24 h. Ideally, additional and earlier time points should have been included for optimal detection of all cytokines at both the mRNA and protein level.

Cytokines can be used as biomarkers to evaluate immune responses. Although additional investigations with larger sample sizes are necessary, this study underscores the viability of employing the bovine bead-based multiplex and qPCR, utilizing the provided primers, for assessing cytokine levels in reindeer samples. For cattle, there are multiple commercial methods and tools available on the market. By knowing which bovine methods that can be successfully used on reindeer, the repertoire of effective ways to survey reindeer health may be expanded. These methods have the potential to provide valuable insights into the health and disease dynamics in wild and semi-domesticated *Rangifer* spp. in rapidly changing northern ecosystems.

## Conclusions

Knowledge about the immune system in reindeer is scarce and there are no specific immunological commercial tools available to study reindeer immunology. The findings of this study show that the MILLIPLEX^®^ Bovine Cytokine/Chemokine multiplex assay is effective in measuring the concentrations of IL-10, IL-17, TNF-α, and IFN-γ in reindeer samples. IL-8 is also detected with this multiplex assay, but high basal levels and challenges with converting MFI to concentration should be considered. The primers used in the study appear promising for measuring IL-8, IL-10, IL-17, TNF-α, IFN-γ and β2M gene expression via qPCR in reindeer. While further investigations with larger sample sets are necessary, this study highlights the potential of the multiplex analysis method and qPCR as diagnostic tools for assessing cytokines in reindeer.

## Prior publication

Data included in this article have previously been published in Norwegian in the form of the master’s thesis, *Diagnostiske verktøy for måling av cytokiner hos rein (Rangifer tarandus tarandus)* (English title: *Diagnostic tools for measuring cytokine levels in reindeer (Rangifer tarandus tarandus)*), in the Norwegian University of Life Sciences (NMBU’s) Open Research Archive, Brage. Details from the previous inoculation experiments (ORFV and CvHV2) from which some of the samples were obtained can be found in the original publications [28, 29].

## Supplementary Information

Additional file 1.

Additional file 2.

Additional file 3.

## Data Availability

The datasets used and/or analysed during the current study are available from the corresponding author on reasonable request.

## Abbreviations

ASFV: African swine fever virus
BLAST: Basic local alignment search tool
BTV: Bluetongue virus
β2M: Beta-2-microglobulin
cDNA: Complementary DNA
ConA: Concanavalin A
CV: Coefficient of variation
CvHV2: Varicellovirus cervidalpha2, formerly known as cervid herpesvirus 2
EDTA: Ethylenediamine tetra-acetic acid
EHV-1: Equine herpesvirus 1
ELISA: Enzyme-linked immunosorbent assay
HSV: Herpes simplex virus
IFN: Interferon
IP-10: IFN-γ-inducible protein 10
IKC: Infectious keratoconjunctivitis
IL: Interleukin
IL-36RA: Interleukin 36 receptor antagonist
LPS: Lipopolysaccharide
LOQ: Limit of quantification
MAFFT: Multiple sequence alignment and fast Fourier transform
MCP-1: Monocyte chemoattractant protein 1
MFI: Median fluorescent intensity
MSA: Multiple sequence alignment
NCBI: National Center for biotechnology information
NK: Natural killer
NMBU: Norwegian university of life sciences
NVI: Norwegian veterinary institute
ORFV: Orf virus
PBMCs: Peripheral blood mononuclear cells
PBS: Phosphate-buffered saline
PHA: Phytohaemagglutinin
p.i.: Post inoculation
PMA-I: Phorbol myristate acetate (PMA) and ionomycin (I)
RPMI: Roswell park memorial institute
SD: Standard deviation
PE: Phycoerythrin
TNF: Tumor necrosis factor
UiT: UiT The Arctic university of Norway
VEGF-A: Vascular endothelial growth factor A
qPCR: Real-time polymerase chain reaction
