# Supplementary material for: Measuring cytokines in Eurasian tundra reindeer (Rangifer tarandus tarandus) with a bovine bead-based multiplex immunoassay and real-time PCR
Source: Acta Vet Scand. 2025 Jun 18;67:34. doi: 10.1186/s13028-025-00819-4 (PMC12175305; doi:10.1186/s13028-025-00819-4)
Supplement: Supplementary file 1 — Additional file 1. [file 13028_2025_819_MOESM1_ESM.docx]

**Additional file 1. Protein levels of cytokines after mitogen stimulation of PBMCs.** Median fluorescent intensity (MFI) of cytokines in supernatants from unstimulated and stimulated peripheral blood mononuclear cells (PBMCs) from three reindeer, measured using MILLIPLEX® Bovine Cytokine/Chemokine multiplex assay. (a) PBMCs were stimulated for 6 hours with phorbol myristate acetate and ionomycin (PMA-I), lipopolysaccharide (LPS), or no mitogen. (b) PBMCs were stimulated for 24 hours with PMA-I, LPS, phytohaemagglutinin (PHA), concanavalin A (ConA), or no mitogen.

**a**

**b**
