# Supplementary material for: Measuring cytokines in Eurasian tundra reindeer (Rangifer tarandus tarandus) with a bovine bead-based multiplex immunoassay and real-time PCR
Source: Acta Vet Scand. 2025 Jun 18;67:34. doi: 10.1186/s13028-025-00819-4 (PMC12175305; doi:10.1186/s13028-025-00819-4)
Supplement: Supplementary file 2 — Additional file 2. [file 13028_2025_819_MOESM2_ESM.docx]

**Additional file 2**

All sequence alignments between reindeer (Rata) and bovine (Bola) sequences were performed using CLUSTAL format alignment by MAFFT (v7.511) (<https://mafft.cbrc.jp/alignment/server/>). Primer regions are shown using red font and sequence identity is shown using stars below the alignment. Sequence references are as follows:

RataIL6 CAI9160207.1, BolaIL6_NP_776348.1, RataIL8 CAI9172108.1, BolaIL8 NP_776350.1, RataIL10 CAI9179163.1, BolaIL10 NP_776513.1, RataIL17 CAI9168504.1, BolaIL17_ACD81652.1, RataTNFa CAI9168605.1, BolaTNFa NP_776391.2, RataIFNg CAI9167936.1, BolaIFNg_NP_776511.1.

Deduced amino acid sequence alignment of bovine and reindeer Interleukin 6

Fwd Rev

RataIL6 MNSLFTSAFSPLAVSLGLLLVMASAFPTPGLLGEDFKNDTTPSRLLLTTPDKTEALIKHI

BolaIL6 MNSRFTSAFTPFAVSLGLLLVMTSAFPTPGPLGEDFKNDTTPGRLLLTTPEKTEALIKRM

*** *****:*:**********:******* ***********.*******:*******::

RataIL6 VDKISAMRKEICEKNDKCENSKETLAENNLNLPKMEEKDGCFQSGFNQETCLIRSTVGLL

BolaIL6 VDKISAMRKEICEKNDECESSKETLAENKLNLPKMEEKDGCFQSGFNQAICLIRTTAGLL

****************:**.********:******************* ****:*.***

RataIL6 EYQTYLDYLQNEYEGDEENVKDLRSSVRTLLQIMRQKV-NLVTTPTTNLDLLEKMQSSNE

BolaIL6 EYQIYLDYLQNEYEGNQENVRDLRKNIRTLIQILKQKIADLITTPATNTDLLEKMQSSNE

*** ***********::***:***..:***:**::**: :*:***:** ***********

RataIL6 WVKNAKIILILRSLENFLQFSLRAIRMK

BolaIL6 WVKNAKIILILRNLENFLQFSLRAIRMK

************.***************

Deduced amino acid sequence alignment of bovine and reindeer Interleukin 8

Fwd

RataIL8 MTSKLAVALLAAFLLSAALCEAAVLSRMSTELRCQCIKTHSTPFHPKFIKELRVIESGPH

BolaIL8 MTSKLAVALLAAFLLSAALCEAAVLSRMSTELRCQCIKTHSTPFHPKFIKELRVIESGPH

************************************************************

Rev

RataIL8 CENSEIIVKLTNGKEVCLNPKEKWVRKVVEVFVKRAEKQDP

BolaIL8 CENSEIIVKLTNGNEVCLNPKEKWVQKVVQVFVKRAEKQDP

*************:***********:***:***********

Deduced amino acid sequence alignment of bovine and reindeer Interleukin 10

RataIL10 MPSSSALLCCLVFLAGVAASRDASALSDSSCTHFPTSLPNMLRELRAAFGRVKTFFQMKD

BolaIL10 M-HSSALLCCLVFLAGVAASRDASTLSDSSCIHLPTSLPHMLRELRAAFGEAKTFFQMKD

* *********************:****** *:*****:**********..********

Fwd

RataIL10 QLDSLLLTQSLLDDFKGYLGCQALSEMIQFYLEEVMPQAENHGPEIKEHVNSLGEKLKTL

BolaIL10 QLHSLLLTQSLLDDFKGYLGCQALSEMIQFYLEEVMPQAENHGPDIKEHVNSLGEKLKTL

**.*****************************************:***************

Rev

RataIL10 RLRLRRCHRFLPCENKSKAVEHVKSVFSKLQERGVYKAMSEFDIFINYIETYTTMKMEN

BolaIL10 RLRLRRCHRFLPCENKSKAVEKVKRVFSELQERGVYKAMSEFDIFINYIETYMTTKMQK

*********************:** ***:*********************** * **::

Deduced amino acid sequence alignment of bovine and reindeer Interleukin 17

RataIL17 MASMRTSSMSLLLLLSLVALVKAGVIIPQSPGCPPTEDKNFPQHVRVNLNIVNRNTNSRR

BolaIL17 MASMRTSSMSLLLLLSLVALVKAGVIIPQSPGCPPTEDKNFPQHVRVNLNIVNRSTNSRR

******************************************************.*****

Fwd Rev

RataIL17 PTDYYKRSTSPWTLHRNEDPERYPSVIWEAKCSHSGCINAEGKVDHHMNSVTIQQEILVL

BolaIL17 PTDYHKRSTSPWTLHRNEDPERYPSVIWEAKCSHSGCINAEGKVDHHMNSVTIQQEILVL

****:*******************************************************

RataIL17 RREPRHCPHSFRLEKMLVAVGCTCVTPIVRHVA

BolaIL17 RRESQHCPHSFRLEKMLVAVGCTCVTPIVRHLA

***.:**************************:*

Deduced amino acid sequence alignment of bovine and reindeer TNF alpha

Fwd Rev

RataTNFa MSTKSMIRDVELAEEELSKKAGGPQGSRSCLCLSLFSFLLVAGATTLFCLLHFGVIGPQR

BolaTNFa MSTKSMIRDVELAEEVLSEKAGGPQGSRSCLCLSLFSFLLVAGATTLFCLLHFGVIGPQR

*************** **:*****************************************

RataTNFa EE-SPTGLSINSPLVQTLRSSSQASINKPVAHVVANINAQGQLLWLDSCANALMANGVKL

BolaTNFa EEQSPGGPSINSPLVQTLRSSSQASSNKPVAHVVADINSPGQLRWWDSYANALMANGVKL

** ** * ***************** *********:**: *** * ** ***********

RataTNFa EDNQLVVPTDGLYLIYSQVLFRGQSCPSTPLFLTHTISRIAVSYQTKVNILSAIKSPCHR

BolaTNFa EDNQLVVPADGLYLIYSQVLFRGQGCPSTPLFLTHTISRIAVSYQTKVNILSAIKSPCHR

********:***************.***********************************

RataTNFa ETPEWAEAKPWYEPIYQGGVFQLEKGDRLSAEINLPDYLDYAESGQVYFGIIAL

BolaTNFa ETPEWAEAKPWYEPIYQGGVFQLEKGDRLSAEINLPDYLDYAESGQVYFGIIAL

******************************************************

Deduced amino acid sequence alignment of bovine and reindeer Interferon gamma

RataIFNg MKYTSYFLALQLCVLLGFSGSYGQGPFFKEIENLKEYFNASSPDVAEGGPLFIEILKNWK

BolaIFNg MKYTSYFLALLLCGLLGFSGSYGQGQFFREIENLKEYFNASSPDVAKGGPLFSEILKNWK

********** ** *********** **:*****************:***** *******

RataIFNg EESDRKIIQSQIVSFYFKLFENFKDNQVIQRSVDIIKQDMFQKFLNGSSEKLEDFKKLIQ

BolaIFNg DESDKKIIQSQIVSFYFKLFENLKDNQVIQRSMDIIKQDMFQKFLNGSSEKLEDFKKLIQ

:***:*****************:*********:***************************

Fwd Rev

RataIFNg IPVDDLQIQRKAINELIKVMNDLSPKSNLRKRKRSQNLFRGRRASM

BolaIFNg IPVDDLQIQRKAINELIKVMNDLSPKSNLRKRKRSQNLFRGRRAST

*********************************************
