# Supplementary material for: Measuring cytokines in Eurasian tundra reindeer (Rangifer tarandus tarandus) with a bovine bead-based multiplex immunoassay and real-time PCR
Source: Acta Vet Scand. 2025 Jun 18;67:34. doi: 10.1186/s13028-025-00819-4 (PMC12175305; doi:10.1186/s13028-025-00819-4)

**Additional file 3**

Products from PCRs performed with complementary DNA (cDNA) from phorbol myristate acetate and ionomycin (PMA-I) stimulated reindeer peripheral blood mononuclear cells (PBMCs) using primers for interferon-gamma (IFN-γ), tumor necrosis factor-alpha (TNF-α), interleukin (IL)-8, IL-10, IL-17, and beta-2-microglobulin (β2M) and the KAPA HiFi HotStart ReadyMix kit were analysed by 2% agarose gel electrophoresis and visualized using the Azurec150 Gel Imaging System.


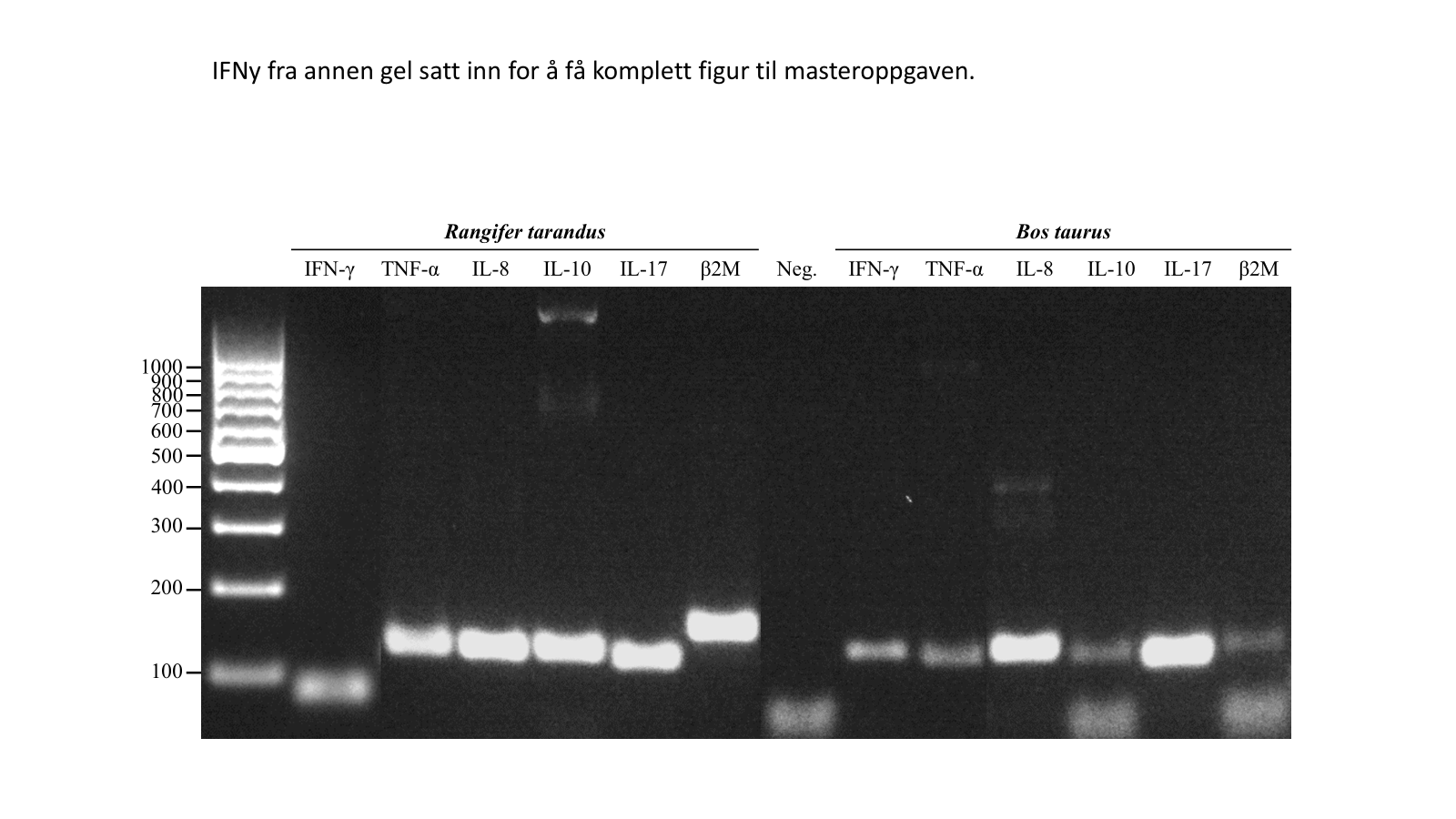

Supplement: Supplementary file 3 — Additional file 3. [file 13028_2025_819_MOESM3_ESM.docx]
